# Chronic Inflammatory Demyelinating Polyneuropathy (CIDP): Overview, Treatment, and a Case Study

**DOI:** 10.7759/cureus.47475

**Published:** 2023-10-22

**Authors:** Madhusudhan Ponnala, Bryan Mullen, Khalid Nawab, Shakir Ullah, Shahbaz Khan, Fayaz Ali

**Affiliations:** 1 Internal Medicine, Penn State Holy Spirit Hospital, Camp Hill, USA; 2 Internal Medicine, Charles R. Drew University, Los Angeles, USA; 3 Emergency Department, Category D Hospital Nawagai, Bajaur, PAK; 4 Internal Medicine, Ayub Medical College, Abbottabad, PAK

**Keywords:** immune globulins, plasmapheresis, cerebrospinal fluid proteins, electrodiagnosis, chronic inflammatory demyelinating poly(radiculo)neuropathy

## Abstract

Chronic inflammatory demyelinating polyneuropathy (CIDP) is an uncommon immune-mediated neuropathy with an often unknown etiology. Patients typically present with gradual muscle weakness, sensory loss, and reduced deep tendon reflexes. Diagnostic challenges persist due to the absence of specific lab findings and definitive criteria. Treatment commonly involves glucocorticoids, IVIG, or plasma exchange, with varied long-term outcomes.

We aim to elucidate the diagnostic complexities and treatment modalities associated with chronic CIDP through a comprehensive review of a patient's clinical presentation, diagnostic work-up, and therapeutic interventions.

A 70-year-old female with a complex medical history, including dermatomyositis and IgG subclass deficiency, presented with progressive lower extremity weakness and numbness. Initial workup including MRI and CT scans were inconclusive. She was diagnosed with CIDP based on electromyography (EMG)/nerve conduction studies and cerebrospinal fluid (CSF) analysis. Plasma exchange (PLEX) treatment was initiated but led to multifocal cerebral infarcts, complicating her course. Subsequent rounds of PLEX alongside dual antiplatelet therapy showed no adverse neurological events and yielded minimal to moderate improvement in her mobility. The patient was discharged to an inpatient rehabilitation center for continued care. Elevated WBCs and other abnormal lab results were monitored throughout, underscoring the need for a multidisciplinary approach in complex cases like this one.

Our comprehensive overview of CIDP and its diagnostic and treatment complexities underscores the challenges clinicians face in both accurate diagnosis and effective management. The multifaceted approach - spanning history-taking, electrodiagnostic studies, and advanced imaging - highlights the necessity for a nuanced, evidence-based practice. The variability in treatment outcomes emphasizes the need for personalized medicine and continuous research to optimize therapeutic strategies. Given the inconclusive nature of some diagnostic tools and the variable treatment responses, there remains a clear need for ongoing study and long-term follow-up to further refine our understanding and management of CIDP.

## Introduction

Chronic inflammatory demyelinating polyneuropathy (CIDP) is an uncommon immune-mediated neuropathy affecting peripheral nerves and nerve roots. The cause of CIDP is unknown in the majority of cases. Patients with CIDP typically present with a gradual-onset, progressive, fairly symmetric muscle weakness with absent or depressed deep tendon reflexes, as well as sensory loss, with weakness being the most prominent symptom [1]. Symptom progression or relapses must be present for at least eight weeks. Constipation and urinary retention are autonomic symptoms that may occur in more severe cases. Laboratory studies are utilized to test for disorders that are associated with or mimic CIDP, but there are no specific lab findings that diagnose CIDP [2]. Electrodiagnostic testing (electromyography (EMG) and nerve conduction studies) reveal evidence of primary demyelination. Cerebrospinal fluid analysis can be helpful when the clinical and electrophysiologic findings are not definitive [3]. Nevertheless, there is no gold-standard set of diagnostic criteria for the diagnosis of CIDP [1]. Treatment for CIPD includes glucocorticoids, intravenous immune globulin (IVIG), or plasma exchange. The long-term prognosis of CIDP patients is favorable for the majority of patients, but some patients will still require chronic immune treatments and others will suffer chronic disabilities [4].

## Case presentation

A 70-year-old, right-handed female with a past medical history significant for dermatomyositis, IgG subclass deficiency, inflammatory arthritis (unspecified), chronic back pain, hyperlipidemia, hypertension, hypothyroidism (Hashimoto’s disease), hypertension, obstructive sleep apnea, depression, generalized anxiety disorder, gastroesophageal reflux disease, and obesity Class III (BMI = 40.4 kg/m2) was brought to Holy Spirit emergency department (ED) on 10/21/2022 with complaints of ambulatory dysfunction and bilateral lower extremity numbness. After a thorough review of her medical record, she reported some subjective left leg weakness and increased fatigue symptoms at a rheumatology appointment in April 2022. She then presented for an acute emergency visit with her family doctor in August 2022 due to left leg and back pain. At this time the patient denied tingling and numbness. However, she stated the problem was becoming persistent and the pain, which she described as aching and deep, was gradually progressing and was aggravated by increased activity. She was treated with a high-dose corticosteroid taper and one-week physical therapy (PT), which did not improve her symptoms. The patient had several falls over the next few days and blood work was completed by her family doctor on 9/14/2022. The blood work results showed an elevated white blood cell (WBC) count, so her family doctor advised her to go to the ED. When she arrived at the ED on 9/15/2022 she complained that her leg weakness was progressing, and she felt like a nerve in her back was "getting pinched" and felt "buzzing" in her bilateral lower extremities. She was found to have a urinary tract infection (UTI) and was subsequently treated with intravenous (IV) antibiotics and transitioned to oral antibiotics. An extensive work-up including labs and imaging (MRI and CT scan of the spine and brain) did not reveal acute abnormalities that could explain her symptoms. The patient had several falls during the hospital course and was discharged to an inpatient PT center on 9/19/2022. The patient participated in two weeks of inpatient PT. She then had an epidural steroid injection on 10/7/2022 and was evaluated at an outpatient neurosurgical clinic on 10/10/2022. During the neurosurgery visit the patient said she felt slightly more ambulatory since the epidural injection but did not feel the numbness and tingling had improved. She mentioned at this point the numbness and tingling were present all the time and did not worsen with activity or rest. The patient was using a wheelchair and ‘rollator’ ambulatory device at the time of the visit. She then went to an outpatient neurology clinic on 10/21/2022 and underwent an electromyography (EMG) and nerve conduction study. The EMG/nerve conduction study showed severe acute sensorimotor polyneuropathy with active denervation and demyelination consistent with lumbosacral plexopathy or a demyelinating disease so the neurologist advised the patient to go to the hospital to have a pelvic CT/ultrasound scan and CSF lumbar puncture performed. Pelvic CT and ultrasound images were obtained in the emergency department, which did not reveal a mass that could be causing a lumbosacral plexopathy. Interventional radiology successfully performed the lumbar puncture on 10/22/2022. The CSF analysis (Table 1) revealed an increase in protein and IgG with a slight elevation of WBCs. There was also a slight elevation in red blood cells (RBCs), but a correction in protein and WBCs was not necessary since the RBC value was only 87 cells/mm3.

**Table 1:** Cerebrospinal Fluid (CSF) Analysis

	Result	Lab Reference Range
Color of Fluid	Colorless	
Protein	140 mg/dL (high)	15-45
Glucose	63 mg/dL	45-70
White Blood Cells	15 cells/mm^3^ (high)	0 cells/mm^3^
Xanthochromia	Negative	Negative
Lymphocytes	14.1 cells/mm^3 ^(high)	0 cells/mm^3^
Monocytes	0.9 cells/mm^3 ^(high)	0 cells/mm^3^
Red Blood Cells	87 cells/mm^3^	0 cells/mm^3^
Albumin	97.8 mg/dL (high)	8.0-42.0 mg/dL
IgG Index	2.10 (high)	<0.66
IgG Synthesis Rate	Plus 123.8	Minus 9.9-3.324
IgG, CSF	31.8 mg/dL (high)	0.8-7.7 mg/dL

Lab work at admission revealed an elevated WBC count, platelet count, anion gap, and increased immature granulocytes. Serum protein, CO2, alkaline phosphatase, and sodium were all decreased.

Urinalysis revealed moderate leukocyte esterase. The patient continued to have urinary retention, which she mentioned she experienced for the past month, so a Foley catheter was placed. Urine culture was growing Staphylococcus species, but not Staph. aureus, so IV vancomycin was prescribed. IV vancomycin was discontinued on 11/1/2022 when the urinalysis had a normal WBC count. The patient had also experienced constipation for the past month. The patient was afebrile at hospital admission and throughout her hospital course. Every system of the physical examination was non-contributory except for the neurological and musculoskeletal exam. Significant musculoskeletal and neurological exam findings included decreased strength in bilateral hip flexors, knee extension, and dorsi- and plantar flexion. Areflexia was demonstrated in the bilateral patella and Achilles tendons. Severely decreased sensation to light touch in bilateral lower extremities from toes to thighs was noted. The patient had moderately impaired vibration sense at the toes, ankles, and knees bilaterally. The gait assessment was deferred because the patient was unable to ambulate. No monoclonal or paraprotein was seen in serum protein electrophoresis (SPEP) labs (Table 2).

**Table 2:** Serum Plasma Electrophoresis (SPEP) Results

Fractions	%	g/dL	Ref Range (g/dL)
Protein total		5.6	5.4 – 8.0
Albumin	63.2	3.5	3.4-5.2
M. Protein Alpha 1	5.6	0.3	0.2-0.4
M. Protein Alpha 2	14.2	0.8	0.4-0.9
M. Protein Beta	10.1	0.6	0.5-1.0
M Protein Gamma	6.9	0.4	0.7-1.5

**Figure 1:**
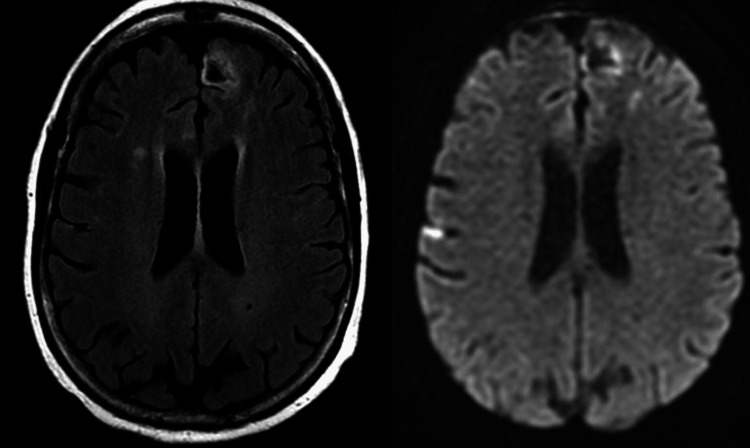
Magnetic resonance imaging (MRI) without contrast 10/28/2022 showing acute ischemic stroke Left: fluid-attenuated inversion recovery (FLAIR). The MRI shows that the largest diffusion restriction focus is 3.5 cm in the left frontal lobe. Right: diffusion-weighted image (DWI) MRI showing corresponding 3.5 cm diffusion restriction in the left frontal lobe.

Based on the neurological examination and EMG/nerve conduction study, the probable working diagnosis was determined to be CIDP. The patient had a history of an allergic reaction to IVIG used to treat a previous dermatomyositis flare-up and recently failed high-dose steroids, so plasma exchange (PLEX) therapy was the treatment option the clinical team and patient decided to initiate. The first therapeutic PLEX occurred on 10/26/2022 and the patient tolerated the procedure well. However, the patient had a transient episode of slurred speech and expressive aphasia on 10/27/2022 (approximately 16 hours after the PLEX), and rapid response was called. There were no focal deficits appreciated on neurological examination and a head CT did not show signs of hemorrhage. A follow-up MRI (Figure 1) on 10/28/2022 demonstrated numerous foci of diffusion restriction involving the frontal and parietal lobes bilaterally consistent with multifocal infarcts in the anterior circulation distributions which were new since the prior brain MRI (9/17/2022).

The PLEX therapy was then discontinued, and an extensive work-up was initiated to explain the ischemic stroke occurrence after PLEX. The work-up included a transesophageal echocardiogram (TEE), ultrasound of the bilateral lower extremities, ultrasound of the bilateral carotid arteries, and wide-ranging bloodwork to assess hypercoagulability, antibodies, and inflammatory markers. The TEE was essentially unremarkable with no thrombus in the atria or at the tip of the catheter used for PLEX, a normal left ventricular ejection fraction, no pulmonary hypertension, no intracardiac shunt, and no significant valvular stenosis or regurgitation. There was no evidence of deep venous or superficial venous thrombosis in the bilateral lower extremities on ultrasound. There was no significant plaque or flow-limiting stenosis of either carotid artery on ultrasound. The bloodwork results are outlined in Table 3 below.

**Table 3:** 10/28/2022 Labs MTHFR Interpretation: This individual is homozygous for the C677T variant and negative (normal) for the A1298C variant in the MTHFR gene. This genotype is common in the general population. Current literature does not support a significant association of this result with CAD, venous thromboembolism, or recurrent pregnancy loss.

		Result	Lab Reference Range
Coagulation Labs	INR	1.1	0.9-1.1
	PT	14.3	12-14.2
	Fibrinogen	203 mg/dL (low)	208-435 mg/dL
	Prothrombin G20210A fact II mutation	Negative	
	Lupus anticoagulant screen	Not detected	
	Dilute Russell viper venom time (DRVVT Screen)	29 seconds	Normal low ≤ 45 seconds
	Methylenetetrahydrofolate reductase (MTHFR)	Positive*	
	Viscosity (serum)	1.5	1.4-1.8
	Partial thromboplastin time -lupus anticoagulant (PTT-LA) screen	30 seconds	Normal low ≤ 40 seconds
Cardiac/Lipids	Homocysteine	5.9 umol/L	5.0-13.9 umol/L
	Total cholesterol	89 mg/dL	≤199 mg/dL
	LDL cholesterol	42 mg/dL	0-99 mg/dL
	HDL cholesterol	24 mg/dL	41 to not specified mg/dL
	Triglycerides	117 mg/dL	≤149 mg/dL
Rheumatology	Cardiolipin antibody, IgG	<2.0 GPL-U/mL	<20 GPL-U/mL (antibody not detected)
	Cardiolipin antibody, IgA	<2.0 GPL-U/mL	<20 GPL-U/mL (antibody not detected)
	Cardiolipin antibody, IgM	<2.0 GPL-U/mL	<20 GPL-U/mL (antibody not detected)
	B2 glycoprotein I IgG	<2.0 unit/mL	<20 unit/mL (antibody not detected)
	B2 glycoprotein IgA	<2.0 unit/mL	<20 unit/mL (antibody not detected)
	B2 glycoprotein IgM	<2.0 unit/mL	<20 unit/mL (antibody not detected)
	Phosphatidyl serine IgG	< 9 units	≤ 30 units
	Phosphatidyl serine IgM	< 9 units	≤ 30 units
	Anti-SSA antibody	< 1.0	<1.0 negative
	Antineutrophil cytoplasmic antibodies (ANCA) screen	Negative	
	C-reactive protein (CRP)	6.14 mg/dL (high)	≤ 0.49 mg/dL

**Figure 2:**
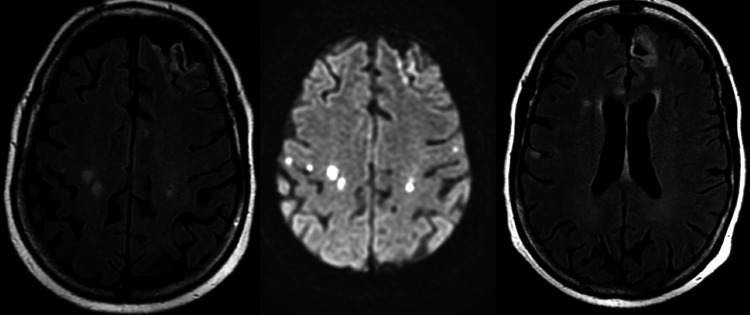
Non-contrast MRI 10/31/2022 Left: FLAIR MRI showing new acute areas of cerebral ischemia most predominantly in the centrum semiovale of both of the cerebral hemispheres Middle: DWI MRI showing corresponding areas of ischemia of bilateral centrum semiovale Right: FLAIR MRI demonstrating old infarct (10/28/2022) with encephalomalacia in the anterior left frontal lobe

The patient had a second transient episode of slurred speech and expressive aphasia on 10/30/2022 (approximately 85 hours after the initial PLEX) and rapid response was called. There were no focal deficits appreciated on neurological examination. The patient reported no new episodes of weakness or numbness. She was still experiencing weakness and numbness in her bilateral lower extremities to the same degree when she presented to the hospital. A head CT did not show signs of hemorrhage. A follow-up MRI on 10/31/2022 demonstrated new acute areas of cerebral ischemia most predominantly in the centrum semiovale of both cerebral hemispheres at the level of the central sulcus bilaterally. The old infarcts in the left frontal lobe from the MRI brain scan on 10/28/22 were also seen (Figure 2).

The patient was already taking aspirin 81 mg daily, and now was started on Plavix 75 mg daily as dual antiplatelet therapy. A CT angiogram of the head was completed on 11/1/2022 to evaluate for any signs of intracranial disease and showed no definitive evidence of a new infarct since the MRI on 10/31/2022. The patient was working with physical therapy (PT) every day during her hospital course, and the PT note on 11/1/22 stated the patient ambulated with her walker a distance of three feet, a minimal improvement since she presented to the hospital. After a discussion with the patient about restarting therapeutic PLEX the patient and spouse decided that would be the plan to potentially continue to see improvements in her mobility. The second, third, fourth, and fifth rounds of therapeutic PLEX took place on 11/2/2022, 11/4/2022, 11/7/2022, and 11/9/2022, respectively.

The patient was continued on dual antiplatelet therapy with aspirin 81 mg daily and Plavix 75 mg daily from 11/1/2022 to 11/11/2022 (patient’s discharge date). Coagulation (PT, INR, and fibrinogen), complete blood cell count, and chemistry profile labs were monitored throughout the PLEX rounds. The significant abnormalities of these labs include consistently low sodium, hemoglobin, and CO2. The patient did not have any transient episodes of speech disturbances or stroke-like symptoms after PLEX rounds 2 through 5. No adverse events were reported after PLEX rounds 2 through 5, other than the patient "feeling cold" during the PLEX. A peripheral blood smear was collected and analyzed on 11/8/2022 which revealed a few teardrop cells and slight burr cells, which were non-specific and non-significant. During the last several days the patient was in the hospital (11/8-11/11), she was able to go from a sitting to a standing position with minimal assistance and using a rolling walker. Additionally, the patient was able to stand and walk short distances with a rolling walker and assistance from her spouse and therapist, displaying minimal to moderate improvement. The patient was discharged on 11/11/22 to an inpatient rehabilitation center. The rest of this paper will focus on the diagnostic modalities and therapy of CIDP, and how they relate to the patient in this case.

## Discussion

Neuropathies can be complex to diagnose because there are over 100 causes of neuropathy [1]. As a syndrome with typical and atypical cases, CIDP has been a difficult disorder to diagnose and treat. Over-reliance on the subjective patient-reported perception of treatment benefit, liberal electrophysiologic interpretation of demyelination, and placing overstated importance on mild or moderate cytoalbuminologic dissociation (CSF finding of elevated protein without pleocytosis) are some of the diagnostic errors leading to misdiagnosis [2]. The pathophysiologic basis for CIDP has not been established, contributing to the challenges in dealing with these patients. The diagnosis of CIDP is based on clinical, electrodiagnostic, and supportive criteria. Although there are no standard criteria used in the diagnosis of CIDP, the European Academy of Neurology (EAN)/Peripheral Nerve Society (PNS) guidelines have been validated in studies to have a sensitivity of 83% and specificity of 96% [3].

When approaching a patient with polyneuropathy, there are parts of the history and physical examination that can help the clinician select a few of the most likely diagnoses. The duration of symptom development is important to categorize the neuropathy as acute or chronic. The EAN/PNS requires symptom progression or relapses that have to be present for at least eight weeks to be defined as chronic, as seen with CIDP [4]. Conversely, Guillain-Barré syndrome (GBS) is an acute inflammatory demyelinating polyneuropathy (AIDP) that reaches its symptom nadir within four weeks of onset. Regarding muscle strength, demyelinating polyneuropathies such as GBS and CIDP are characterized by proximal dominant weakness, since multiple roots are often affected by conduction block. Assessment of deep tendon reflexes is an important part of the physical examination when determining if neuropathy is central or peripheral. Hyporeflexia or areflexia indicates a peripheral source, whereas hyperreflexia usually indicates a central pathology. Additionally, it may be helpful to determine the extent of motor and sensory nerve involvement since some forms of neuropathy are predominantly sensory which can be seen in amyloid, diabetes, thiamine deficiency, and sarcoid. GBS and CIDP are predominantly motor neuropathies. This is not always straightforward in clinical life because the majority of neuropathies are mixed, but it may help in the diagnosis on a case-by-case basis. The patient in this case had mixed motor and sensory neuropathy, which did not rule out CIDP as a diagnosis.

Labs are helpful in the work-up to rule out other causes of peripheral neuropathy such as diabetes mellitus, Vitamin B12 deficiency, connective tissue disorders, and various malignancies. MRI of the spine with contrast is the main imaging modality that can aid in the correct diagnosis of CIDP. The patient in this case had an overabundance of labs drawn during her hospital course which did not point to a different diagnosis than CIDP. MRI showing contrast enhancement of the cauda equina or lumbosacral nerve roots can support the diagnosis of CIDP. Contrast-enhanced MRI of the spine is also helpful in ruling out other diagnoses such as transverse myelitis, multiple sclerosis (MS), or spinal stenosis. The patient in this case did not have spinal MRI findings to support a CIDP diagnosis. However, the patient’s spine MRI also did not show a structural reason (spinal stenosis) for her symptoms and did not have findings consistent with MS or transverse myelitis, so CIDP could still be considered at that point based on the patient’s clinical presentation and EMG results.

Electrophysiologic studies (EMG and nerve conduction studies) are mandatory to make a CIDP diagnosis. The characteristic electrophysiologic features of CIDP include partial conduction block, conduction velocity slowing, prolonged distal motor latencies, delay or disappearance of F waves, and temporal dispersion and distance-dependent reduction of compound motor action potential (CMAP) amplitude. However, electrophysiologic studies have sub-optimal sensitivity and specificity, so their results alone should not define a diagnosis of CIDP [5]. Even though CIDP is a primarily demyelinating disorder, some degree of axonal degeneration is usually present as well [6], which is theorized to be a secondary bystander product of the inflammatory demyelinating process. The patient in this case had prominent evidence of demyelination on EMG and nerve conduction studies, which prompted the neurologist to recommend the patient go to the hospital.

CSF analysis is useful to distinguish immune-mediated neuropathies such as CIPD. CSF protein is elevated (>45 mg/dL) and the CSF white cell count is normal in more than 80% of patients with CIPD [7]. Increased CSF protein without pleocytosis (WBC count <10 cells/mm3) is generally considered as supporting criteria to diagnose CIDP. The patient in this case did have CSF protein elevation (140 mg/dL) but did have a CSF WBC count of 15 cells/mm3. Therefore, the patient did not fall into the criteria to support the diagnosis of CIPD based on the CSF results. However, the WBC count was only five above the supported criteria, so a follow-up CSF outpatient setting is recommended.

The mainstay treatments for CIDP include high-dose corticosteroids, IVIG, and plasmapheresis (PLEX). These three therapies have shown equal efficacy [4]. The choice of therapy is individualized for each patient depending on history, allergies, and previous therapies attempted. At the completion of the treatment trial, objective signs of efficacy should be evident to continue maintenance treatment. It is important to understand that stability in a patient who previously was progressing may indicate a treatment response in CIDP patients. Relapse is a common issue when treating CIDP patients. For PLEX specifically, the most common adverse events reported are urticaria (transfusion reaction), transient hypocalcemia, transient hypotension, headache, and infection related to the catheter access site [8]. To our knowledge, there has been no reported occurrence of ischemic stroke following PLEX therapy. A case-crossover study determined that patients admitted for venous thrombosis embolism (but not acute ischemic stroke or myocardial infarction) were more likely exposed to either IVIG or PLEX during previous admission for neurologic disease [9]. It is difficult to determine if the PLEX therapy was the cause of the patient’s stroke in our case study. However, a very thorough work-up was completed to determine the potential cause of stroke for our patient, which was unrevealing. More data is needed to determine the long-term outcomes and prognosis for CIPD patients. A five-year follow-up study by Kuwabara et al [10]. that analyzed 38 CIDP patients who underwent either high-dose corticosteroids, IVIG, PLEX, or a combination of these therapies showed that 10 patients had complete remission of symptoms with normal nerve conduction studies. However, 23 patients had partial remission with or without needing future immune treatments. The study found that patients with a more favorable long-term prognosis had a subacute onset and symmetrical presentation of symptoms, good response to initial corticosteroid treatment, and nerve conduction abnormalities predominantly in distal nerve terminals, whereas patients who experienced treatment relapse or were refractory to treatment had an insidious onset of symptoms, asymmetrical symptoms, and electrophysiological evidence of demyelination in intermediate nerve segments.

## Conclusions

The complexity of diagnosing and treating CIDP can't be overstated, given the myriad of underlying causes for neuropathies and the non-standardized criteria for diagnosis. The incorporation of a holistic approach encompassing clinical presentation, CSF studies, EMG, nerve conduction studies, and imaging like MRI is paramount for an accurate diagnosis. However, the limitations of each diagnostic modality must be critically acknowledged. While EAN/PNS guidelines have been validated for sensitivity and specificity, they are not without their limitations, particularly when facing atypical presentations as in the case discussed.

The evolving landscape of therapeutic options, including corticosteroids, IVIG, and plasmapheresis, offers clinicians multiple avenues for treatment. Yet, treatment responses can be varied and the risk profile, as evidenced by the patient in this case study who suffered from an ischemic stroke, must be carefully weighed. This individual variability in treatment response highlights the need for personalized medicine approaches in managing CIDP. Continual re-evaluation of diagnostic criteria, regular follow-up CSF studies, and a thorough cardiovascular risk assessment prior to treatment are crucial elements in enhancing patient outcomes.

Moreover, it's imperative to focus on longitudinal studies that address long-term outcomes, in order to better inform therapeutic choices and prognostication. Key characteristics like subacute onset and symmetrical presentation can offer valuable prognostic insights. Further high-quality, evidence-based studies are needed to refine diagnostic and treatment guidelines, minimize misdiagnosis, and improve the long-term outcomes of patients with CIDP.

In summary, CIDP remains a diagnostic and therapeutic challenge requiring a multifaceted, evidence-based approach, underscored by the commitment to continual learning and adaptation as new data becomes available. Given the complexity of the disorder and the significant impact on patients' quality of life, the medical community must remain vigilant in pursuing rigorous, comprehensive research to refine our understanding and management of CIDP.
